# Claudin18.2 defines a prognostically distinct subgroup of intrahepatic cholangiocarcinoma via CD8^+^ T-cell exclusion

**DOI:** 10.3389/fonc.2025.1636367

**Published:** 2025-08-29

**Authors:** Chengui Yu, Yuren Pan, Fuli Li, Zhenyun Guo, Da Xu, Ying Zhu, Baobing Yin

**Affiliations:** ^1^ Department of Hepatopancreatobiliary Surgery, National Medical Center, Binhai Campus of the First Affiliated Hospital, Fujian Medical University, Fuzhou, China; ^2^ Fujian Abdominal Surgery Research Institute, the First Affiliated Hospital, Fujian Medical University, Fuzhou, China; ^3^ Hepatobiliary Surgery, Department of General Surgery, Huashan Hospital & Cancer Metastasis Insititute, Fudan University, Shanghai, China; ^4^ Department of General Surgery, The Fifth People’s Hospital of Wuxi, Wuxi, China

**Keywords:** CLDN18.2, intrahepatic cholangiocarcinoma, prognosis, clinicopathological features, CD8+ T-cell

## Abstract

**Background and purpose:**

Intrahepatic cholangiocarcinoma (ICC) is an aggressive malignancy with limited therapeutic options. Claudin18.2 (CLDN18.2), a tight junction protein aberrantly expressed in gastrointestinal cancers, has not been systematically evaluated in ICC. This study investigates CLDN18.2’s expression, clinical relevance, and interplay with the tumor immune microenvironment (TIME) in ICC.

**Method:**

CLDN18.2 expression was analyzed in 83 ICC and 47 matched non-tumor tissues on tissue microarray sections using immunohistochemistry (IHC). Bioinformatics validation utilized ArrayExpress (E-MTAB-6389) and GEO (GSE119336, GSE107943, GSE89749, GSE32225) datasets. Clinicopathological correlations, survival analysis, and CD8^+^ tumor-infiltrating lymphocytes (TILs) quantification were performed.

**Results:**

CLDN18.2 was exclusively expressed in 24.1% (20/83) of ICC tissues, absent in non-tumor tissues. Positive CLDN18.2 expression correlated with elevated serum CA19-9 (*P* = 0.026), smaller tumor size (*P* = 0.03), unifocality (*P* = 0.03), and higher recurrence (*P* = 0.018). Multivariable analysis identified CLDN18.2 as an independent prognostic factor for reduced overall survival (OS: HR = 2.555, 95% CI = 1.250–5.223, *P* = 0.01) and disease-free survival (DFS: HR = 2.229, 95% CI = 1.125–4.415, *P* = 0.022). Single-sample gene set enrichment analysis (ssGSEA) analysis revealed an inverse correlation between CLDN18 expression and CD8^+^ T cells (*P* = 0.012), while IHC showed a trend toward negative correlation between CLDN18.2 expression and CD8^+^ TILs density (*P* = 0.12). Combined stratification showed optimal OS in CLDN18.2^-^/CD8^high^ patients versus worst outcomes in CLDN18.2^+^/CD8^low^ subgroup (*P* = 0.006).

**Conclusions:**

CLDN18.2 is a tumor-specific prognostic biomarker in ICC, marking aggressive subsets with early recurrence. Combined CLDN18.2/CD8^+^ TILs stratification enhances prognostic precision and suggests synergistic potential for CLDN18.2 targeted therapies with immunomodulation. These findings warrant clinical validation to guide personalized treatment strategies.

## 1 Introduction

Intrahepatic cholangiocarcinoma (ICC) is a highly heterogeneous malignancy originating from the epithelial cells of the intrahepatic bile ducts at grade II or above, and constitutes approximately 10% of primary liver cancers. Its incidence has risen globally, particularly in Asia (1, 2). Although surgical resection remains the sole potentially curative option, most patients present with advanced-stage disease, and only 20–30% are candidates for resection (3). Postoperative recurrence rates remain alarmingly high, with a dismal 5-year survival rate of 30% (4). Patients with recurrent or advanced ICC exhibit poor chemosensitivity. Recent advances in targeted therapies, including inhibitors against isocitrate dehydrogenase (IDH) and fibroblast growth factor receptor (FGFR), have shown limited utility due to low mutation frequencies in ICC populations, particularly among Chinese cohorts (3, 5, 6). Therefore, these challenges underscore the urgent need to identify novel therapeutic targets and develop precision treatment strategies to improve ICC outcomes.

Claudins (CLDNs), a family of 27 transmembrane tight junction proteins, regulate paracellular permeability, maintain epithelial polarity, and modulate signal transduction (7, 8). Claudin18 (CLDN18), encoded by a gene located on chromosome 3q22.3, exists as two splice variants: CLDN18.1 (predominantly expressed in lung alveolar epithelial cells) and CLDN18.2 (primarily restricted to gastric mucosa) (9). CLDN18.2 is a highly selective marker protein with restricted expression in healthy tissues, being expressed only in differentiated gastric mucosal epithelial cells (10). Abnormal expression of CLDN18.2 can lead to structural damage and functional impairment of tissue epithelial and endothelial cells, which is observed in the tumorigenesis process of many malignant tumors (11–13). Emerging CLDN18.2 targeted therapies, such as the monoclonal antibody zolbetuximab, have demonstrated survival benefits in phase III trials (SPOTLIGHT and GLOW) for advanced gastric adenocarcinoma when combined with chemotherapy (14, 15). In addition, preclinical and early-phase trials are further exploring antibody-drug conjugates and CAR-T therapies for CLDN18.2-positive solid tumors, potentially extending therapeutic applicability to ICC (16). However, despite a report suggesting upregulated CLDN18 (encompassing both isoforms) in ICC, the specific biological significance of CLDN18.2, including its associations with clinicopathological features, prognosis, and the tumor immune microenvironment (TIME), remains uncharacterized in ICC (17).

This study aims to: investigate the expression of CLDN18.2 in ICC; analyze the correlation between CLDN18.2 expression and clinicopathological characteristics and prognosis of ICC; and explore its relationship with the TIME in ICC, particularly CD8^+^ tumor-infiltrating lymphocytes (TILs). These investigations aim to seek potential new therapeutic targets and treatment strategies for ICC.

In this study, we used immunohistochemistry (IHC) to evaluate the expression level of CLDN18.2 in 83 ICC specimens and 47 adjacent non-tumor tissues. Multivariate analyses identified independent prognostic factors, while TILs quantification elucidated associations between CLDN18.2 and immune contexture.

## 2 Materials and methods

### 2.1 Study population and tissue collection

This retrospective study included 83 ICC specimens and 47 adjacent non-tumor tissues obtained from patients undergoing curative-intent resection at Huashan Hospital, Fudan University between February 2017 and April 2021. All cases were histologically confirmed as ICC and independently validated by two board-certified pathologists. The study protocol adhered to the Declaration of Helsinki and was approved by the Ethics Committee of Huashan Hospital. The clinicopathological features included age, sex, tumor size, tumor number, histological grade, bile duct and perineural invasion, vascular invasion (microvascular/macrovascular), TNM stage (the 8th edition of American Joint Committee on Cancer (AJCC) stage), tumor markers (CA19–9 and CEA), and HBV status. The definition of recurrence relied on comprehensive assessment of imaging, tumor biomarkers and surgical pathology. Follow-up time was calculated from the date of initial surgery to the date of the observed event (death or recurrence), with a follow-up date up to February 2024.

### 2.2 Immunohistochemical staining and evaluation

**Figure 1:**
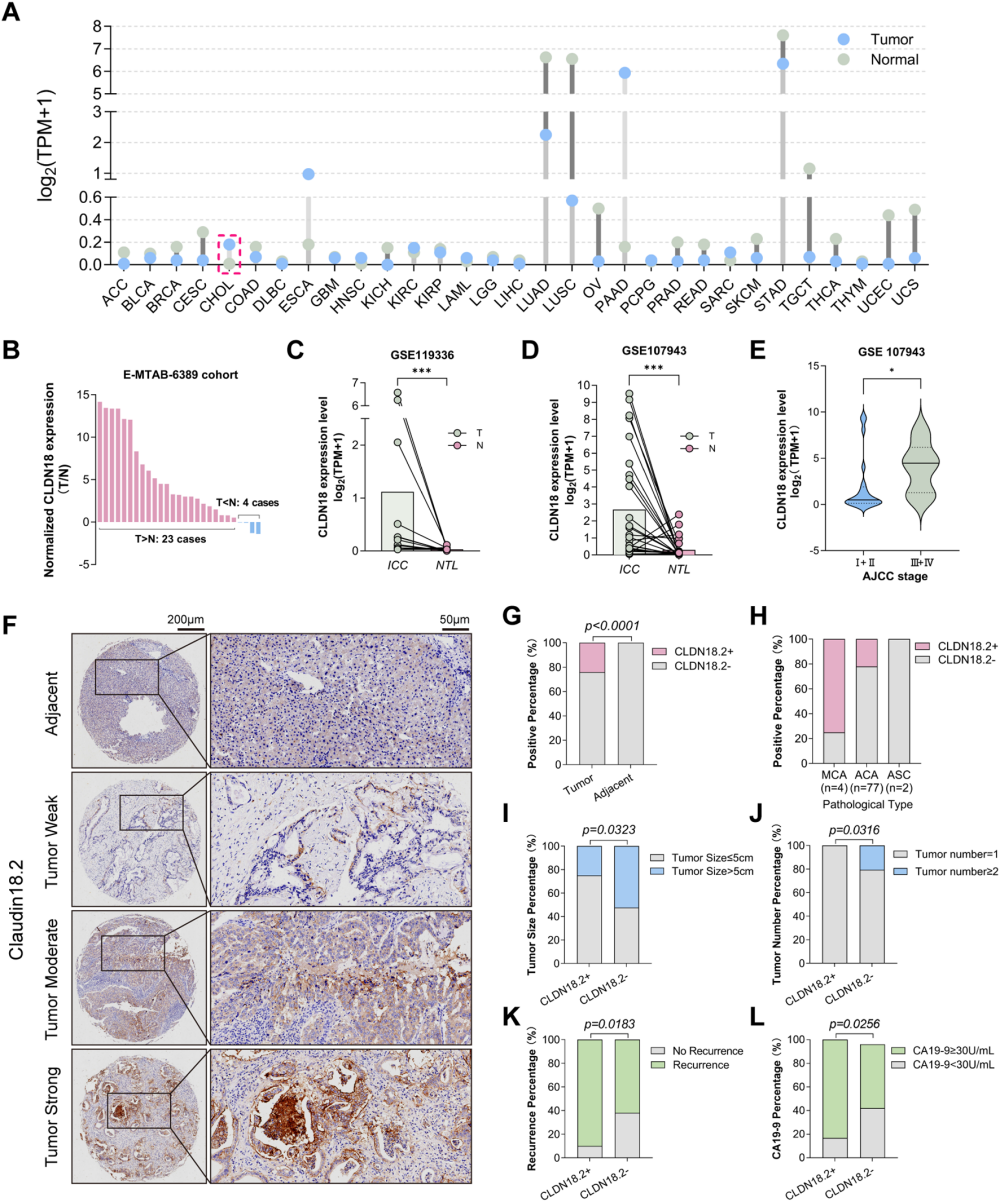
CLDN18.2 expression patterns and clinicopathological correlations in ICC. **(A)** CLDN18 expression in common malignant tumors in the TCGA database. **(B)** In the E-MTAB-6389 cohort, CLDN18 expression was elevated in ICC tumors in 23 paired samples, and decreased in only 4 paired samples. **(C, D)** Expression of CLDN18 in ICC tumors was higher than that in normal tissues, based on the GSE119336 and GSE107943 datasets (*P* < 0.001, Wilcoxon signed-rank test). **(E)** CLDN18 expression level in different ICC AJCC stages, based on the GSE107943 dataset (*P* < 0.05). **(F)** The immunohistochemical staining intensity of CLDN18.2 in ICC tissue and adjacent non-tumor tissues (no staining; weak staining; moderate staining; strong staining). **(G)** Differential expression of CLDN18.2 in tumor and adjacent non-tumor tissues of ICC (*P* < 0.05, Fisher’s exact test). **(H)** Differential expression of CLDN18.2 levels by pathological type. **(I-L)** The proportions of higher serum CA19-9, smaller tumor size, unifocal tumor and recurrence were significantly increased in the CLDN18.2- positive ICC patients (all *P* < 0.05, Fisher’s exact test or Chi-square test).

The tumor tissue samples and adjacent non-tumor tissue samples were fixed in formalin and embedded in paraffin (FFPE). Then the FFPE slices were prepared for tissue microarrays (TMA). IHC staining was performed to analyze the expression of CLDN18.2 and the tumor-infiltrating CD8^+^ T cells using a KFBIO KF-PRO-120 automatic immunostaining device according to the manufacturer’s instructions. Primary antibodies used were anti-CLDN18.2 (Abcam, ab222512, dilution 1:100) and anti-CD8 (Abcam, ab245118, dilution 1:500). Membranous staining of CLDN18.2 in tumor cells was assessed using the H-score system. The H-score was calculated using the formula: H-score = ∑ (*i* × *p*), where *i* represents the staining intensity (0 = no staining, 1 = weak, 2 = intermediate, 3 = strong; **Figure 1F**) and *p* represents the proportion of positively stained tumor cells (0 = 0%, 1 = 1-25%, 2 = 26-50%, 3 = 51-75%, 4 = 76-100%). The H-scores ranged from 0 to 12, with H-scores ≥1 indicating positive CLDN18.2 expression; H-scores = 0 indicating negative CLDN18.2 expression (18). CD8^+^ tumor-infiltrating lymphocytes were quantified and categorized into high and low groups based on the median count per high-power field.

### 2.3 Bioinformatics analysis

The Cancer Genome Atlas (TCGA), Gene Expression Omnibus (GEO) and ArrayExpress data were used to analyze the expression of the CLDN18 gene in intrahepatic cholangiocarcinoma and adjacent non-tumor tissue, assess the impact of CLDN18 on survival in ICC patients and explore the relationships between CLDN18 expression and tumor-infiltrating cells. The analysis of CLDN18 expression and immune cell infiltration was performed using single-sample gene set enrichment analysis (ssGSEA) and TIMER method in the IOBR R package (19).

### 2.4 Statistical analysis

The statistical data were analyzed with IBM SPSS 26.0 for Windows, RStudio (R version 4.5.0) and GraphPad Prism software (Version 9.1). A comparison between groups was made using the Wilcoxon rank-sum, chi-square or Fisher’s exact test for categorical variables and one-sample t-test or one-way ANOVA for continuous variables. Survival analysis was performed using the Kaplan-Meier method and log-rank test. Univariate and multivariate analyses were performed using Cox proportional hazards regression. A *P*-value < 0.05 was considered to be statistically significant. Overall survival (OS) was assessed from the day of first surgery to the date of death. Disease-free survival (DFS) was defined from the day of tumor resection to disease recurrence.

## 3 Results

### 3.1 Clinicopathological features of ICC patients

The baseline clinicopathological characteristics of 83 ICC patients are summarized in **Table 1**. The cohort comprised 57 males (68.7%) and 26 females (31.3%), with a median age of 60.1 years (range: 32–82 years). Tumors ≤ 5 cm in diameter were observed in 45 cases (54.2%), and 69 patients (83.1%) presented with unifocal tumors. Hepatitis B virus (HBV) infection was detected in 47 patients (56.6%), while elevated serum CA19–9 levels were found in 46 of 79 patients (58.2%; data missing for 4 patients). Pathological assessment revealed lymph node metastasis in 18 patients (21.7%). Most ICC cases (65/83; 78.3%) were classified as early-stage ICC (stage I/II according to the 8th edition AJCC TNM staging system), and tumor recurrence occurred in 57 patients (68.7%).

**Table 1:** Clinicopathological characteristics and CLDN18.2 expression in ICC patients.

Characteristics	Total (N=83)	Claudin18.2 expression		χ2	P value
		Positive	Negative		
Age (years)				0.019	0.89
≤ 65	55 (66.3%)	13 (65.0%)	42 (66.7%)		
>65	28 (33.7%)	7 (35.0%)	21 (33.3%)		
Sex				2.29	0.13
Female	26 (31.3%)	9 (45.0%)	17 (27.0%)		
Male	57 (68.7%)	11 (55.0%)	46 (73.0%)		
Tumor size (cm)				4.59	0.032*
≤ 5	45 (54.2%)	15 (75.0%)	30 (47.6%)		
>5	38 (45.8%)	5 (25.0%)	33 (52.8%)		
Tumor number					0.032*
1	70 (83.1%)	20 (100.0%)	50 (79.4%)		
≥2	13 (16.9%)	0 (0.0%)	13 (20.6%)		
Histological grade				H = 0.25	0.62
Well/Moderate	64 (77.1%)	16 (80.0%)	48 (76.2%)		
Moderate-poor	9 (10.8%)	3 (15.0%)	6 (9.5%)		
Poor	10 (12.0%)	1 (5.0%)	9 (14.3%)		
Lymph node metastasis					0.35
Yes	18 (21.7%)	6 (30.0%)	12 (19.0%)		
No	65 (78.3%)	14 (70.0%)	51 (81.0%)		
AJCC TNM stage				1.07	0.30
I/II	65 (78.3%)	14 (70.0%)	51 (81.0%)		
III/IV	18 (21.7%)	6 (30.0%)	12 (19.0%)		
MVI				1.60	0.21
M0	48 (57.8%)	14 (70.0%)	34 (54.0%)		
M1/M2	35 (42.2%)	6 (30.0%)	29 (46.0%)		
^a^CA199				6.04	0.026*
Positive	48 (58.2%)	15 (83.3%)	33 (54.1%)		
Negative	31 (41.8%)	3 (16.7%)	28 (45.9%)		
HBV				2.97	0.085
Positive	47 (56.6%)	8 (40.0%)	39 (61.9%)		
Negative	36 (43.3%)	12 (60.0%)	24 (38.1%)		
Microbiliary invasion				0.17	0.68
Yes	30 (36.1%)	8 (40.0%)	22 (34.9%)		
No	53 (63.9%)	12 (60.0%)	41 (65.1%)		
Perineural invasion				0.07	0.78
Yes	27 (32.5%)	7 (35.0%)	20 (31.7%)		
No	56 (67.5%)	13 (65.0%)	43 (68.3%)		
Cirrhosis					0.43
Yes	10 (12.0%)	1 (5.0%)	9 (14.3%)		
No	73 (88.0%)	19 (95.0%)	54 (85.7%)		
^a^CEA				0.96	0.76
Positive	24 (30.4%)	6 (33.3%)	18 (29.5%)		
Negative	55 (69.6%)	12 (66.7%)	43 (70.5%)		
^a^ AFP					0.66
Positive	7 (8.9%)	2 (11.1%)	5 (8.2%)		
Negative	72 (91.1%)	16 (88.9%)	56 (91.8%)		
Recurrence					0.018*
Yes	57 (68.7%)	18 (90.0%)	39 (61.9%)		
No	26 (31.3%)	2 (10.0%)	24 (38.1%)		

N, total number of cases; AJCC, American Joint Committee on Cancer.

TNM, tumor, node, metastasis; HBV, hepatitis B virus; CA199, Carbohydrate antigen199; MVI, Microvascular Invasion; CEA, Carcinoembryonic antigen; AFP, Alpha fetoprotein.

^a^4 cases were loss of tumor marker data.

*P < 0.05.

### 3.2 CLDN18.2 is specifically expressed in ICC

Due to the difficulty of distinguishing between CLDN18 isoforms in bioinformatics transcriptomic databases, we used CLDN18 to represent CLDN18.2 expression in ICC. The results of the bioinformatics analysis demonstrated significantly elevated CLDN18 mRNA levels in ICC versus adjacent non-tumor tissues (*P* < 0.05; **Figures 1A-D**), with positive correlation between CLDN18 expression and advanced AJCC stage (*P* < 0.05; **Figure 1E**). Representative images of CLDN18.2 immunohistochemical staining in ICC are shown in **Figure 1F** (detailed explanations are provided in the Materials and Methods section). Immunohistochemical analysis of TMAs revealed CLDN18.2 positivity (H-score ≥ 1) exclusively in ICC specimens (20/83, 24.1%), absent in adjacent non-tumor tissues (0/47; *P* < 0.0001; **Figures 1G, H**). Subtype-specific analysis showed positive expression in 75% of mucinous carcinomas (3/4), 22% of adenocarcinomas (17/77), and no expression in adenosquamous carcinomas (0/2; **Figure 1H**).

### 3.3 Relationship between clinicopathological features and the expression of CLDN18.2 in ICC

CLDN18.2 expression showed significant correlation with smaller tumor size (*P* = 0.032), unifocal tumors (*P* = 0.032), elevated serum CA19-9 (*P* = 0.026), and higher recurrence rates (*P* = 0.018; **Table 1**, **Figures 1I-L**). No associations were observed with AJCC stage, lymph node metastasis, histological grade, microvascular invasion, microbiliary invasion or perineural invasion (**Table 1**).

### 3.4 Prognostic significance of CLDN18.2 in ICC

**Figure 2:**
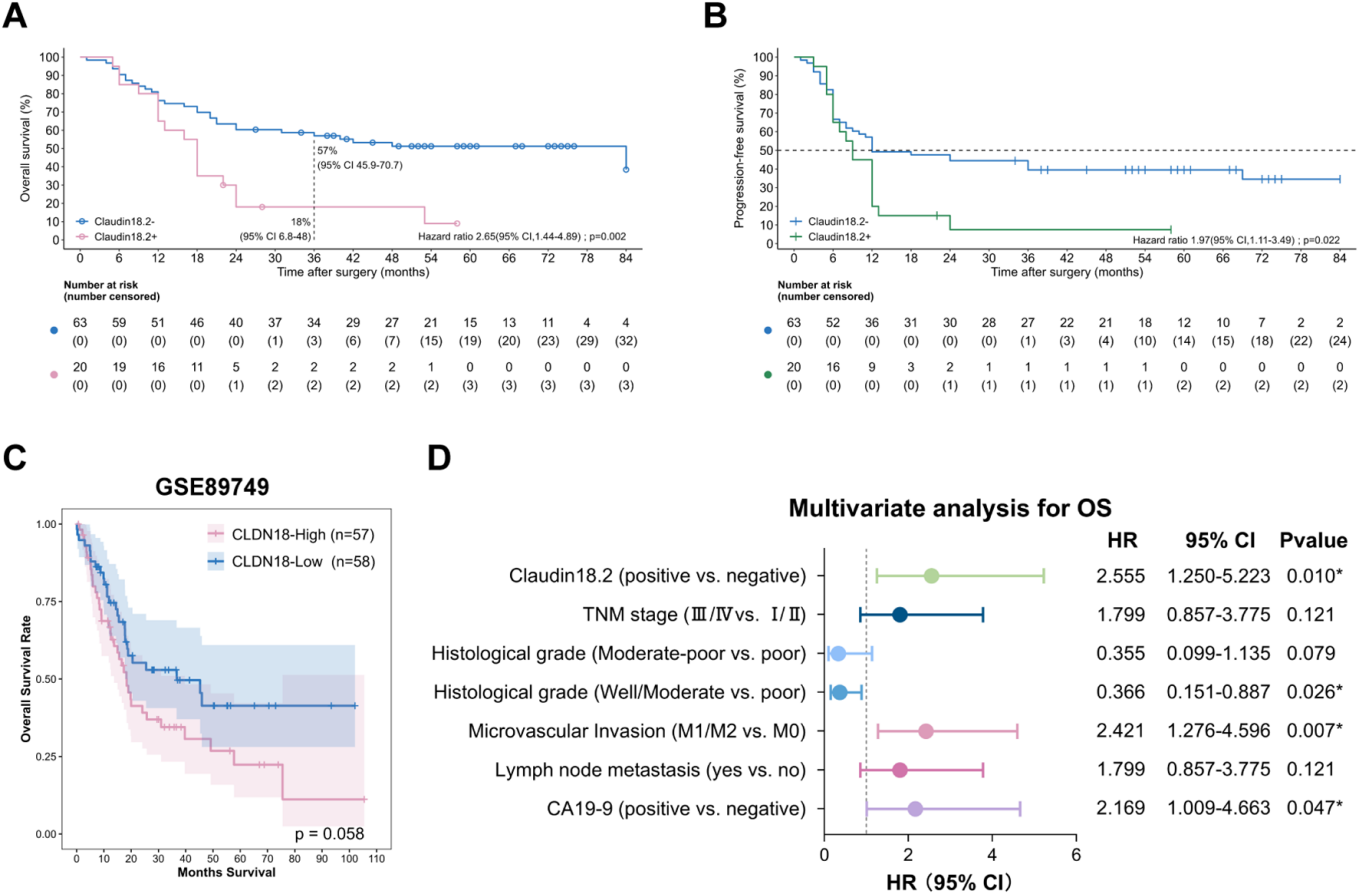
Prognostic significance of CLDN18.2 expression in ICC patients. **(A)** Kaplan–Meier OS curves of ICC patients with different CLDN18.2 expression levels in tumor tissues (log-rank test; *P* = 0.002). **(B)** Kaplan–Meier DFS curves of ICC patients with different CLDN18.2 expression levels in tumor tissues (log-rank test; *P* = 0.02). **(C)** The OS with different CLDN18 expression levels in ICC patients, analyzed with the GSE89749 dataset. **(D)** Forest plot of the results of the multivariate COX regression model for exploring potential risk factors for OS in ICC patients.

In our cohort, with a median follow-up of 34.5 months (range: 1–84 months), Kaplan-Meier analysis demonstrated significantly shorter OS (median OS: 18 vs. 84 months; *P* = 0.002) and disease-free survival (DFS) (median DFS: 9 vs. 12 months; *P* = 0.02) in CLDN18.2-positive versus CLDN18.2-negative groups (**Figures 2A, B**). Similarly, transcriptomic analysis (GSE89749) indicated ICC patients with high CLDN18 expression had a trend toward poorer overall survival (OS) (*P*=0.058; **Figure 2C**).

Cox regression analysis was employed to identify independent risk factors that may influence the prognosis of ICC. For OS, univariate Cox analysis identified positive CLDN18.2 expression (HR = 2.545, 95% CI = 1.383-4.682; *P* = 0.003), serum CA19–9 elevation, advanced TNM stage, poor histological grade, microvascular invasion (MVI) and lymph node metastasis as significant factors (**Table 2**, all *P* < 0.05). Subsequent multivariable Cox analysis confirmed that positive CLDN18.2 expression remained significantly associated with OS (HR = 2.555, 95% CI = 1.250-5.223; *P* = 0.01). Additionally, serum CA19–9 elevation, poor histological grade and MVI were significantly associated with OS (**Figure 2D**, all *P* < 0.05). These findings indicate that positive CLDN18.2 expression is an independent prognostic factor for ICC.

**Table 2:** Univariate analysis of prognostic factors(OS) for ICC.

Parameters	HR	95% CI	P value
Age (years) (> 65 vs. ≤ 65)	0.690	0.369-1.291	0.246
Sex (male vs. female)	0.805	0.445-1.455	0.472
HBV	1.079	0.609-1.915	0.794
CA199	3.487	1.710-7.110	0.001*
CEA	1.638	0.885-3.032	0.116
TNM stage (III/IV vs. I/II)	2.165	1.129-4.151	0.020*
Histological grade			0.010*
(Moderate-poor vs. poor)	0.376	0.124-1.138	0.083
(Well-Moderate vs. poor)	0.312	0.147-0.660	0.002*
MVI (M1/M2 vs. M0)	2.600	1.459-4.635	0.001*
Lymph node metastasis	2.165	1.129-4.151	0.020*
Bile duct invasion	1.372	0.766-2.458	0.288
Perineural invasion	1.542	0.858-2.773	0.148
Tumor number (≥ 2 vs. 1)	0.885	0.396-1.977	0.766
Tumor size(cm) (> 5 vs. ≤ 5)	1.594	0.904-2.811	0.107
Cirrhosis	0.739	0.292-1.868	0.522
Claudin18.2	2.545	1.383-4.682	0.003*

a) *Statistically significant (*P* < 0.05).

For DFS, univariate Cox analysis showed that positive CLDN18.2 expression (HR = 1.865, 95% CI = 1.053-3.304; *P* = 0.033), large tumor size, serum CA19–9 elevation, advanced TNM stage, poor histological grade, MVI and lymph node metastasis were all significantly associated with disease recurrence. The other clinicopathologic features were not significantly associated with recurrence. Further multivariable Cox regression identified positive CLDN18.2 expression (HR = 2.229, 95% CI = 1.125-4.415; *P* = 0.022), large tumor size, advanced TNM stage, poor histological grade, MVI and lymph node metastasis as independent prognostic factors for disease recurrence (**Table 3**, all *P* < 0.05).

**Table 3:** Cox regression analyses of disease-free survival for ICC.

	Univariable analysis		Multivariable analysis	
Parameters	HR (95% CI)	P value	HR (95% CI)	P value
CA199	2.350 (1.311-4.213)	0.004*	1.517 (0.789-2.920)	0.212
TNM stage (III/IV vs. I/II)	2.594 (1.419-4.742)	0.002*	2.612 (1.313-5.197)	0.006*
Histological grade		0.049*		0.409
(Moderate-poor vs. poor)	0.637 (0.237-1.716)	0.373	0.606 (0.199-1.844)	0.378
(Well-Moderate vs. poor)	0.414 (0.199-0.860)	0.018*	0.549 (0.228-1.323)	0.181
MVI (M1/M2 vs. M0)	2.358 (1.384-4.019)	0.002*	2.086 (1.157-3.761)	0.014*
Lymph node metastasis	2.594 (1.419-4.742)	0.002*	2.612 (1.313-5.197)	0.006*
Claudin18.2	1.865 (1.053-3.304)	0.033*	2.229 (1.125-4.415)	0.022*
Tumor size (cm) (> 5 vs. ≤ 5)	1.832 (1.087-3.087)	0.023*	2.443 (1.357-4.400)	0.003*

a) *Statistically significant (*P* < 0.05).

### 3.5 Association between CLDN18.2 and CD8^+^ TILs in ICC

**Figure 3:**
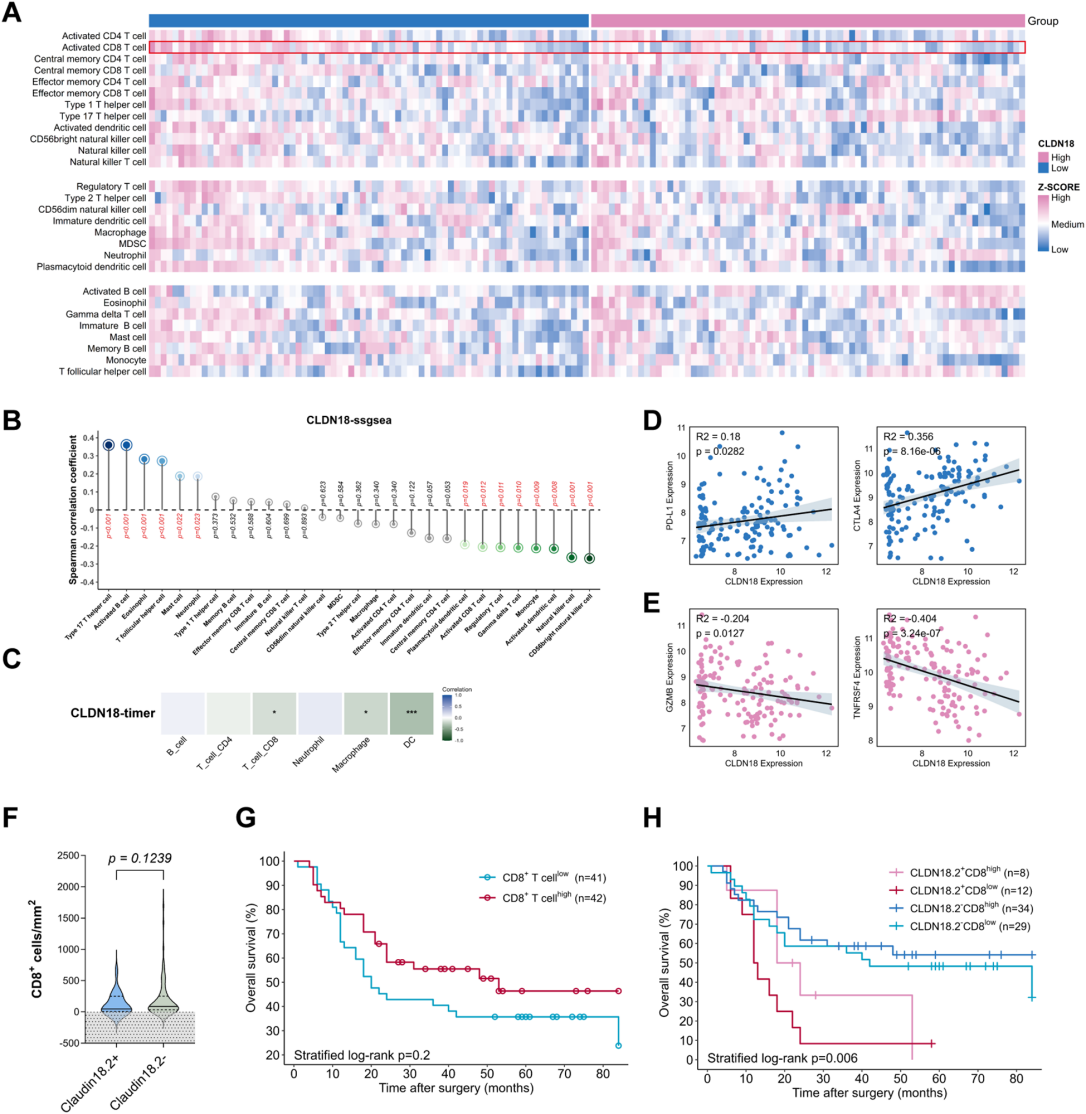
Association between CLDN18.2 expression and tumor immune microenvironment. **(A-C)** Relationship between CLDN18 expression levels and immune-infiltrating cells based on the GSE32225 dataset. **(D, E)** Relationship between CLDN18 expression levels and CD8^+^ T cell-related immune molecules (all *P* < 0.05). **(F)** The density of CD8^+^ TILs in CLDN18.2^+^ ICC compared with CLDN18.2- ICC (*P* > 0.05; Mann Whitney test). **(G)** Kaplan–Meier OS curves of ICC patients with high/low densities of CD8^+^ TILs in tumor tissues (*P* > 0.05; log-rank test). **(H)** Kaplan–Meier OS curves of ICC patients based on CLDN18.2 expression in combination with densities of CD8^+^ TILs (*P* < 0.05; log-rank test).

Using the ssGSEA algorithm, analysis of CLDN18 expression and tumor-infiltrating immune cells based on GEO dataset GSE32225 revealed inverse correlations between CLDN18 expression and CD8^+^ TILs (**Figures 3A, B**), which was consistent with outcomes using TIMER algorithm (**Figure 3C**). Furthermore, CLDN18^high^ ICC tumors exhibited downregulated CD8^+^ TILs activation markers (TNFRSF4, GZMB) and upregulated immune checkpoints (PD-L1, CTLA-4) (**Figures 3D, E**, all *P* < 0.05). We therefore further assessed the expression levels of tumor-infiltrating CD8^+^ TILs in ICC samples using IHC. Given the limited sample size, we found that the CLDN18.2 expression showed a weak negative correlation with the density of tumor-infiltrating CD8^+^ TILs (**Figure 3F**, *P* = 0.12). The CD8^+^ TILs density alone had no significant effect on the prognosis of ICC patients (**Figure 3G**, *P* = 0.2). However, combining CLDN18.2 expression with CD8^+^ TILs resulted in notable differences in the Kaplan-Meier survival analysis. Combined stratification showed optimal OS in CLDN18.2^-^/CD8^high^ patients versus worst outcomes in CLDN18.2^+^/CD8^low^ subgroup (**Figure 3H**, *P* < 0.05).

## 4 Discussion

Intrahepatic cholangiocarcinoma (ICC) represents a highly aggressive malignancy with a 5-year survival rate of 5–10%, significantly worse than hepatocellular carcinoma (20, 21). Despite advances in targeted therapies (e.g., IDH/FGFR inhibitors), <10% of Chinese ICC patients harbor actionable mutations, underscoring the need for novel therapeutic strategies. CLDN18.2, a tight junction protein physiologically restricted to gastric mucosa, has emerged as a promising pan-cancer target due to its tumor-selective overexpression in gastric, pancreatic, and esophageal malignancies (10, 13, 16, 22–24). Our study provides evidence of CLDN18.2 expression in ICC, with 24.1% positivity (20/83) exclusively in tumor tissues, aligning with prior transcriptomic reports but resolving isoform-specific ambiguity through CLDN18.2-specific IHC.

Previous research has shown that the CLDN18.2 protein is expressed exclusively in differentiated gastric mucosal epithelial cells in normal tissues, but is retained during malignant transformation (10). In addition to *in situ* expression in gastric cancer, CLDN18.2 has been found to be aberrantly expressed in many malignant tumors (13). This makes it an attractive target for cancer therapy (25). Aya Shinozaki et al. revealed that Claudin18 was expressed in ICC tissue, but not in normal biliary epithelial tissue (17). This is consistent with our bioinformatics analysis results. However, the immunohistochemical analysis of previous study employed a non-specific polyclonal antibody against CLDN18.2, which is a drawback due to the cross-reactivity of CLDN18.1. In the present study, we used an antibody specific for CLDN18.2. This is the first report on the expression of CLDN18.2 in patients with ICC. Our findings confirm exclusive CLDN18.2 expression in 24.1% of ICC cases with complete absence in normal adjacent tissue, highlighting its therapeutic target potential. These findings demonstrate that CLDN18.2 is specifically expressed in ICC, which is consistent with the results of previous studies. This implies that CLDN18.2 may act as a potential immunotherapeutic target for ICC patients. Currently, a number of pharmaceutical agents and therapeutic modalities targeting CLDN18.2 are undergoing clinical trials for the treatment of various solid tumors (26), with encouraging outcomes, particularly in gastric cancer (14, 15). Recent phase 1 trials demonstrate that Claudin18.2-specific CAR T cell therapy (satri-cel/CT041) is entering clinical exploration for gastrointestinal cancers, showing promising activity with a 50% objective response rate in biliary tract cancer patients and overall response rates of 42.2% across various digestive system malignancies (27, 28). Nevertheless, it is important to recognize that the presence of a target expression in a tumor does not necessarily indicate that the patient will derive benefit from the corresponding targeted drug. Therefore, further clinical trial studies should be designed to confirm the efficacy of CLDN18.2 targeted drugs in ICC patients.

Mechanistically, CLDN18.2-positive ICCs demonstrated aggressive clinical behavior, correlating with elevated serum CA19-9, early recurrence, and poor prognostic value (OS: HR = 2.555, 95% CI = 1.250-5.223, *P* = 0.01). These findings extend observations by Takasawa et al., who implicated CLDN18-driven EGFR/ERK activation in cholangiocarcinoma progression (29). Notably, 90% (18/20) of CLDN18.2-positive cases recurred post-resection, suggesting CLDN18.2 may mark a subset of ICCs with inherent metastatic propensity, even among early-stage tumors.

To our knowledge, the local immune response to tumors is primarily mediated by TILs (30). One of the most important cell types is CD8^+^ TILs, which exert anti-tumor immune effects by directly killing the tumor cells (31). The high density of tumor-infiltrating CD8^+^ T cells in tumor tissue is consistently associated with a favorable prognosis in numerous malignant neoplasms (32–34). While transcriptomic data (GSE32225) indicated CLDN18^high^ ICCs exhibit CD8^+^ T-cell exclusion and upregulated immune checkpoints (PD-L1/CTLA-4), our IHC cohort showed weak direct correlation, possibly due to the small sample size. This discrepancy may reflect spatial heterogeneity or post-transcriptional regulation. Crucially, combined CLDN18.2/CD8^+^ T-cell stratification identified distinct prognostic subgroups, CLDN18.2^+^/CD8^low^ ICC patients had the worst prognosis (*P* = 0.006). This suggests synergistic therapeutic potential for CLDN18.2 targeted agents (e.g., zolbetuximab) combined with PD-1/CTLA-4 blockade, warranting preclinical validation.

Our study has several limitations. First, the data presented in this study was collected from a single medical center, and the number of samples is relatively limited. Therefore, the results may not be fully representative of the wider ICC patient population. Large-scale, multi-center studies are required for further validation in the future. Second, while both previous and our current study have identified obvious intra-tumor heterogeneity in CLDN18.2 expression (35), the use of tissue microarrays as a proxy resulted in an incomplete assessment of CLDN18.2 expression. Multiple tissue cores or whole tumor sections could be used in the future to better assess CLDN18.2 expression in ICC. Third, athough this study provides valuable insights through IHC, it did not utilize multiplex immunohistochemistry (mIHC), which limits the ability to analyze the spatial distribution and interactions of multiple immune markers simultaneously. Future studies could incorporate mIHC for more detailed immune profiling and a better understanding of marker interactions. Fourth, lack of standardized CLDN18.2 scoring criteria for ICC, requiring harmonization with gastric cancer thresholds (H-score ≥1) until disease-specific guidelines emerge. Further studies are required to ascertain this criterion.

## 5 Conclusion

In conclusion, this study establishes CLDN18.2 as a tumor-specific biomarker in ICC, expressed in 24.1% of ICC patients with absent normal tissue expression. CLDN18.2 positivity independently predicts early recurrence and reduced overall survival, while combined CLDN18.2/CD8+ T-cell stratification enhances prognostic precision. These findings position CLDN18.2-directed therapies (e.g., antibody-drug conjugates, CAR-T) as viable strategies for ICC, particularly when integrated with immune checkpoint inhibitors. Prospective trials validating CLDN18.2 as both a predictive biomarker and therapeutic target are urgently needed to translate these insights into clinical practice.

## Data Availability

The raw data supporting the conclusions of this article will be made available by the authors, without undue reservation.
